# Severe wound infection by MRCNS following bilateral inguinal herniorrhaphy

**DOI:** 10.1186/s12879-023-08039-9

**Published:** 2023-02-07

**Authors:** Yao Du, Song Han, Yue Zhou, Hai Feng Chen, Yao Liang Lu, Zhi Yuan Kong, Wei Ping Li

**Affiliations:** 1 Department of General Surgery, The First People’s Hospital of Taicang City, Taicang Affiliated Hospital of Soochow University, Taicang City, 215400 Jiangsu Province China; 2 Department of General Surgery, The First Affiliated Hospital of Nanchang University, Nanchang City, 330006 Jiangxi Province China; 3 Department of Gastroenterology, The First People’s Hospital of Taicang City, Taicang Affiliated Hospital of Soochow University, Taicang City, 215400 Jiangsu Province China

**Keywords:** MRCNS, Wound infection, Hernia, Inguinal

## Abstract

**Background:**

Wound infection after inguinal hernia surgery is not uncommon in the clinical setting. The common microbial aetiology of postoperative inguinal hernia wound infection is Gram-positive bacteria. *Staphylococcus aureus* is a common pathogen causing wound infection while *Staphylococcus epidermidis* and Pseudomonas are rare. *Staphylococcus epidermidis* as a cause of severe wound infection is rarely described in literature. We herein present a case of a 79-year-old man with a rare wound infection after bilateral inguinal herniorrhaphy caused by MRCNS (Methicillin Resistant Coagulase Negative *Staphylococcus*).

**Case presentation:**

We present a case of wound infection accompanied by fever with a temperature of 38.8 °C after bilateral inguinal herniorrhaphy in a 79-year-old man. Bilateral inguinal wounds were marked by redness and swelling, with skin necrosis. In addition, an abscess of approximately 1.5 cm × 1.5 cm was seen on the left wrist. A small amount of gas under the skin in the wound area was observed after pelvic computed tomography (CT) scans. No bacteria were cultured from the inguinal wound discharge, while blood culture detected MRCNS, and *Acinetobacter lwoffi* was cultured from the pus in the left wrist. We chose appropriate antibiotics based on the results of the bacterial culture and the drug susceptibility results. Vacuum assisted closure (VAC) therapy was used after debridement. The patient was discharged after the wounds improved. He was followed up for ten months and showed no signs of complications. We are sharing our experience along with literature review.

**Conclusions:**

We are presenting a rare case of MRCNS wound infection following open inguinal hernia surgery. Although a rarity, clinicians performing inguinal hernia surgery must consider this entity in an infected wound and follow up the patient for complications of MRCNS.

## Background

Inguinal hernia is a mass formed by abdominal visceral organs protruding to the body surface through the inguinal region defect. The lifelong cumulative incidence of inguinal hernia repair in adults is 27–42.5% for men and 3–5.8% for women [1]. Groin hernia repair is one of the most frequently performed surgical procedures worldwide, and hernioplasty probably remains the first choice and most frequently applied surgical method in a majority of cases in many countries [1]. Inguinal hernia repair is considered a clean surgical procedure with a low risk (< 5%) of postoperative wound infection [2, 3]. Even under the most scrupulous aseptic conditions and with a careful technique, postoperative wound infection still presents a very serious problem, and when infection complications occur following inguinal hernia repair, they can be a risk factor for developing a recurrent hernia [4]. Therefore, it is necessary to detect wound infections early and administer timely treatment. Here, we report a rare case of severe wound infection after bilateral inguinal herniorrhaphy due to MRCNS in a 79-year-old man who shared diagnosis and treatment experience.

## Case presentation

**Fig. 1:**
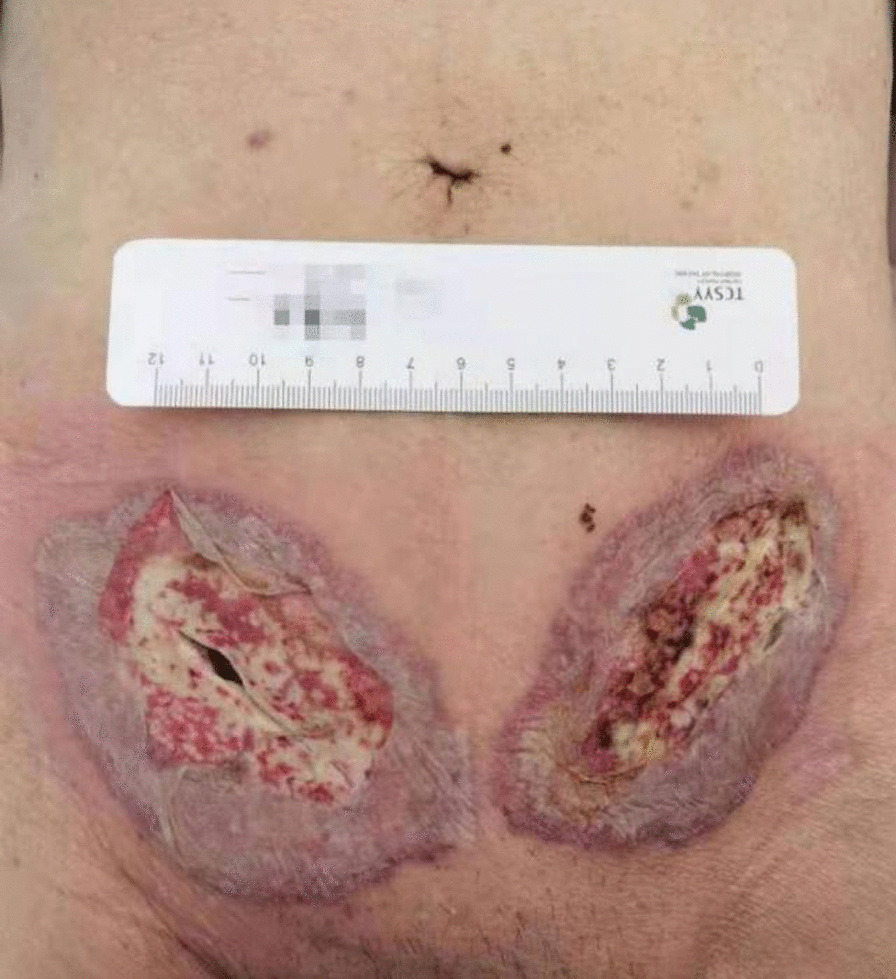
Physical examination showed wounds of approximately 5 cm in length in the bilateral groin areas. The wounds were marked by redness and swelling, and the skin around the wounds were red and white

**Fig. 2:**
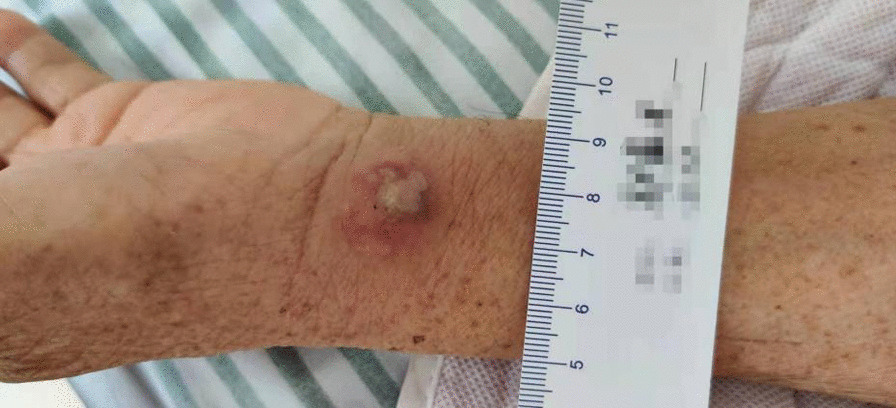
An abscess of approximately 1.5 cm × 1.5 cm was seen on the left wrist without rupture

**Fig. 3:**
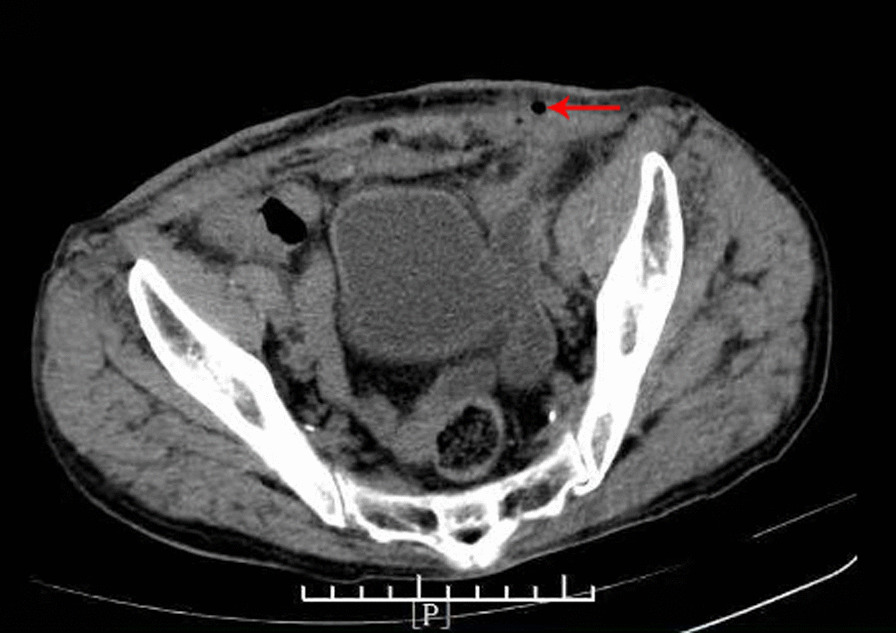
Pelvic CT scans showed that the subcutaneous fat space in the wound area was blurred, a small amount of gas was scattered under the skin in the wound area (arrows), and the fat space in the pelvis was blurred

A 79-year-old Chinese man came to the hospital due to bilateral inguinal reproducible masses he noticed one week prior. The patient had a history of cerebral infarction and prostatic hyperplasia. An open tension-free hernia repair bilateral direct inguinal hernia (Bard Modified Kugel Hernia Patch) was performed on February 4, 2021, and the patch was placed in the preperitoneal space. The patient had no prophylactic antibiotics before surgery and was discharged 2 days after surgery with no swelling at the wound site. On February 8, the patient came to our hospital for symptomatic treatment of wound dressing change and had redness and swelling around the left groin wound, accompanied by a small amount of pale-yellow exudate. On February 11, the patient’s bilateral groin wounds were red and swollen, with skin necrosis. He came to our hospital’s surgical outpatient clinic again, and was admitted to the hospital with wound infection. Measurement of vital signs revealed a body temperature of 38.8 °C, blood pressure of 121/57 mmHg, and a pulse rate of 120 beats/min. Abdominal examination showed no positive signs. Physical examination showed wounds of approximately 5 cm in length in the bilateral groin areas. The wounds were marked by redness and swelling, and the skin around the wounds were red and white with a slightly yellowish exudate (Fig. 1). In addition, an abscess of approximately 1.5 cm × 1.5 cm was seen on the left wrist without rupture (Fig. 2). He was diagnosed with bilateral inguinal wound infection. Pelvic CT scans showed that the subcutaneous fat space in the wound area was blurred, a small amount of gas was scattered under the skin in the wound area, and the fat space in the pelvis was blurred (Fig. 3). The results of laboratory examination were as follows: white blood cell (WBC), 17.4 × 10^9^/L; haemoglobin level, 95 g/L; C-reactive protein (CRP), 200 mg/L; procalcitonin (PCT), 2.610 ng/mL; albumin, 30.7 g/L; potassium, 2.90 mmol/L; sodium, 129.0 mmol/L.

**Fig. 4:**
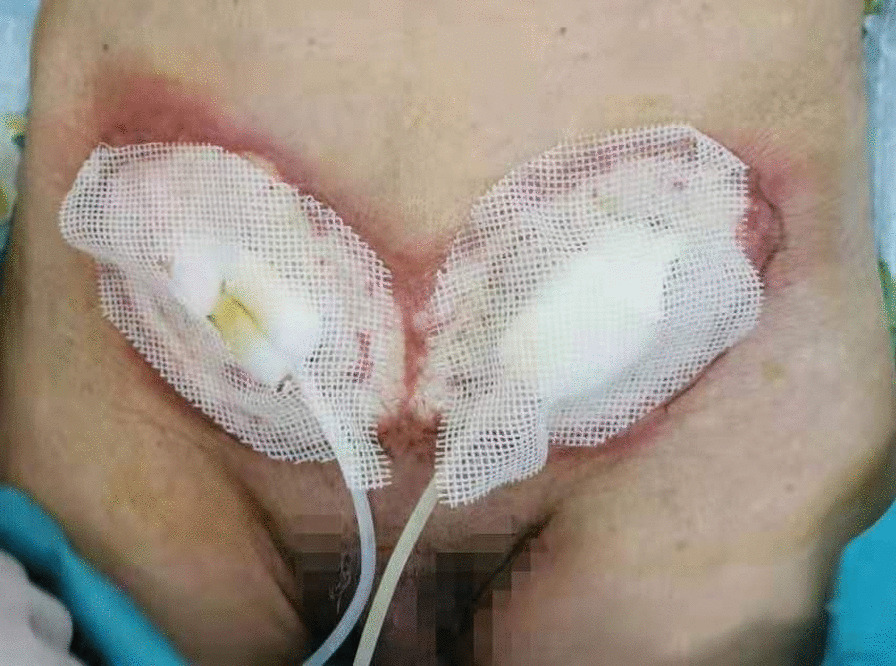
The bilateral inguinal wounds were subjected to a simple VAC device

**Fig. 5:**
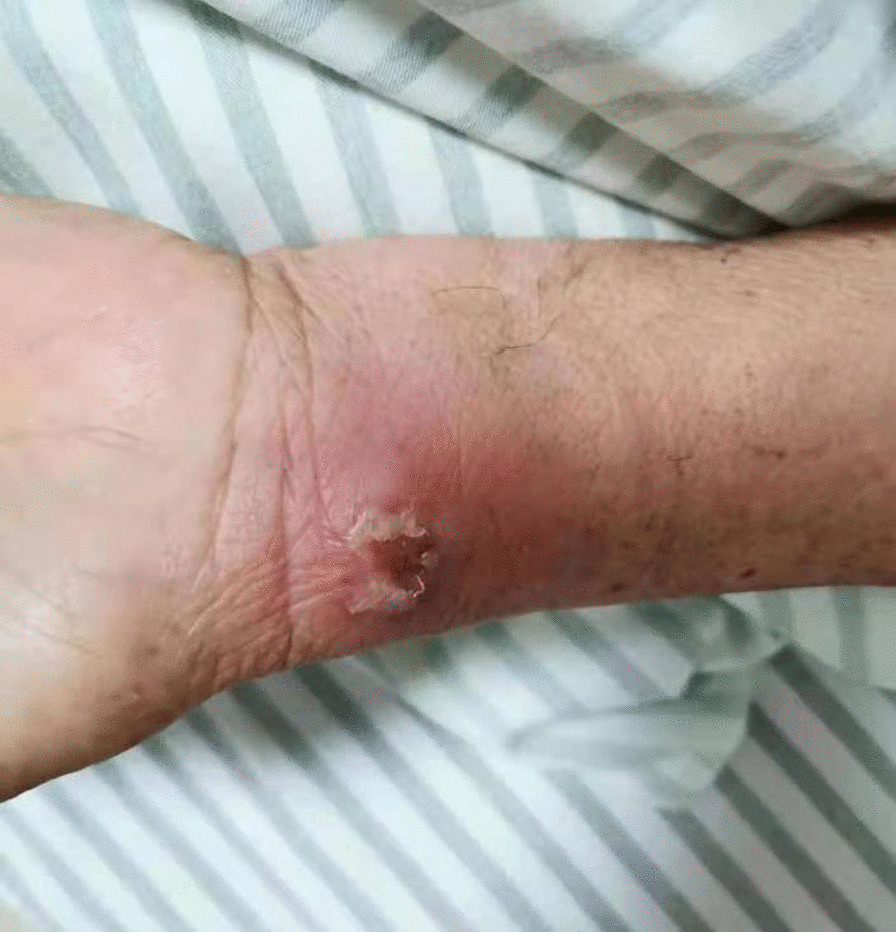
The puncture site of the left wrist had scabbed

**Fig. 6:**
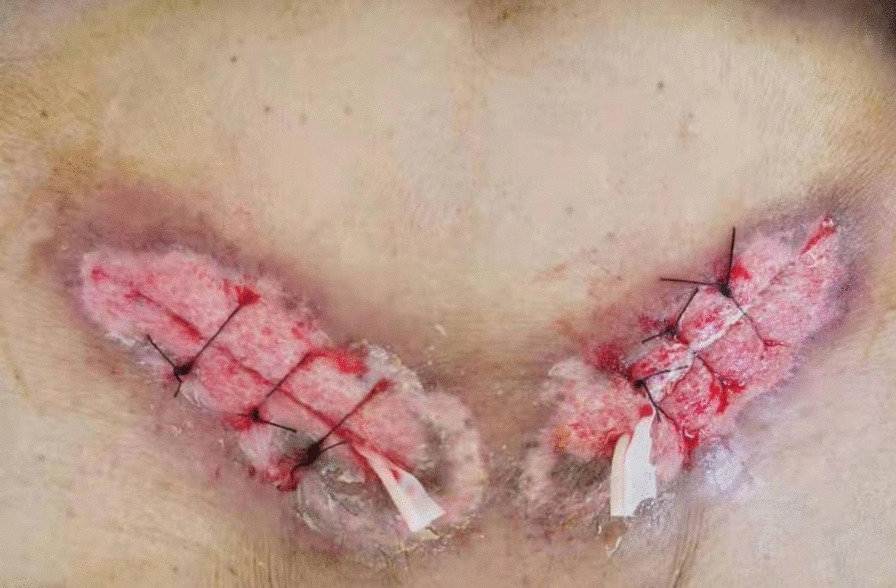
On March 3, the inguinal wound was resutured, and a surgical drain was placed for drainage

Bacterial culture and selection of antibiotics: For the wound infection after bilateral inguinal herniorrhaphy, the patient was given cefoperazone sodium sulbactam sodium and ornidazole for anti-infection supplemented with other symptomatic support treatments. The inguinal wound discharge, left wrist abscess, and blood were sent for bacterial culture on February 12. During treatment, the patient continued to have a fever, the scope of the wound infection continued to expand, and it continued to invade the surrounding epidermis. We considered the possibility of Gram-positive bacterial infection, so the patient’s antibiotics were changed to moxifloxacin and penicillin on February 14. On February 15, normal abdominal wall and spermatic cord tissue were seen after opening the external oblique muscle aponeurosis, without pus, tissue necrosis, and patch exposure. It was considered that wound infection had not significantly affected the patch at present. One day later, the necrotic tissue of the inguinal wound was sent for pathological examination, and the bilateral inguinal wounds were subjected to a simple VAC device (Fig. 4). VAC therapy was used after debridement. On February 17, bacterial culture results showed that no bacteria were cultured from the inguinal wound discharge, while *Staphylococcus epidermidis* (MRCNS) was detected in blood culture, and the pus in the left wrist was cultured with *Acinetobacter lwoffi*. According to the drug susceptibility results, penicillin was replaced with clarithromycin as anti-infection agents on February 17, and the inflammatory indices (WBC, CRP, and PCT) were significantly decreased compared with the previous examination. Through the joint consultation of the Department of Infectious Diseases and the Department of Pharmacopathology with the drug susceptibility results, the patient was placed on imipenem and vancomycin for anti-infection treatment on February 18. The inguinal wound discharge and blood were sent for bacterial culture again on February 20. However, the puncture site of the left wrist had scabbed, and the wound discharge could not be taken for culture (Fig. 5). No bacterial growth was found in the re-examination results, and the patient’s body temperature and inflammatory indices gradually decreased to normal levels. Imipenem was discontinued on February 27. One day later, Vancomycin and VAC were discontinued, and the wound dressings were changed regularly. Linezolid was used to fight infection on March 1. On March 3, the inguinal wound was resutured, and a surgical drain was placed for drainage (Fig. 6). Two days later, the surgical drain was removed. Body temperature monitoring and re-examination of inflammatory indicators were normal, and linezolid was discontinued on March 7.

**Fig. 7:**
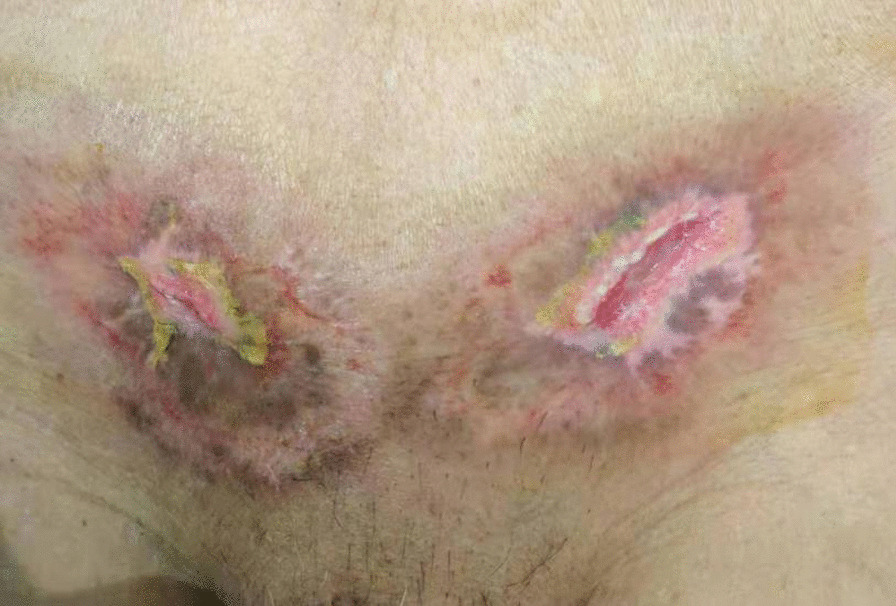
The bilateral groin wounds were almost healed

**Fig. 8:**
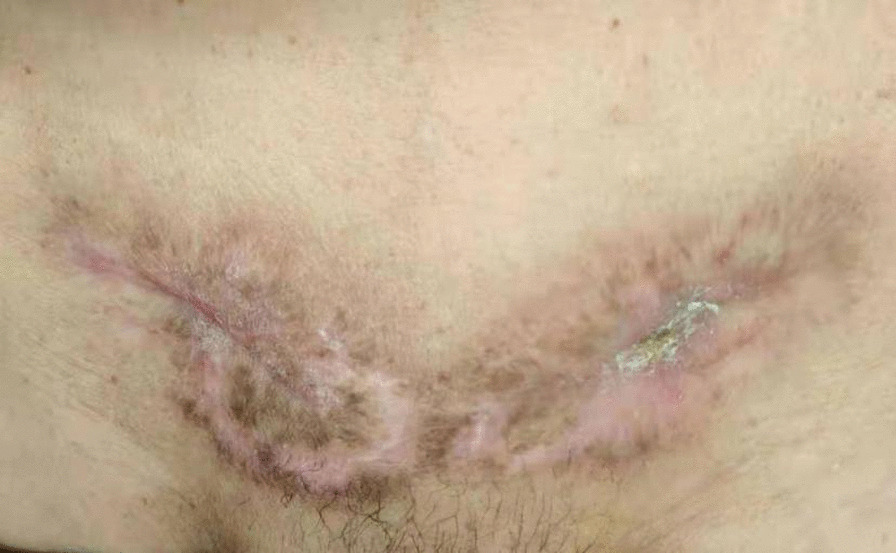
The wound had completely healed

Surgical wound treatment and outcome: Regular wound dressings were performed, but the wound infection developed rapidly and invaded the surrounding tissue in a circular band. Grey‒white necrotic tissue was visible in the skin around the wound after the wound was split. The skin in the middle was purplish red, and the outer skin was red and swollen. The pathological results showed that the wound tissue had hyperplasia of collagen fibres and inflammatory cell infiltration with necrosis. The bilateral groin wound infection was significantly improved after active treatment modalities. The wound sutures were removed on March 17. The bilateral groin wounds were almost healed (Fig. 7) on March 21. Regular wound dressings and outpatient follow-up were continued after the patient was discharged from the hospital. The wound had completely healed (Fig. 8) on April 20. The patient showed no signs of complications after 10 months of follow-up.

## Discussion and conclusions

Wound infection is a common postoperative complication for tension-free inguinal hernia repair. Upon microbiological culture of the wound exudates, most often normal nasal or skin bacteria such as *Staphylococcus aureus* or *Staphylococcus epidermidis* bacterial strains are detected [5, 6]. There are many factors that can cause inguinal hernia wound infection, including patient factors and surgery-related factors, such as length of operation, aseptic conditions, operation room ventilation, and expertise of the surgeon, which can affect postoperative wound infection rates. Sereysky et al. [7] reported that diabetes, BMI ≥ 35 kg/m^2^, and current smoking are significantly associated with an increased odds of surgical site infections (SSIs) after initial, open, reducible inguinal hernia repair in adults with clean surgical sites. For ‘clean’ surgery, such as inguinal hernia repair, antibiotic prophylaxis is not generally recommended, while antibiotic prophylaxis is recommended when prosthetic material is being used or when risk factors are present [8, 9]. However, a randomized controlled trial published by Mazaki et al. indicated that the use of prophylaxis is effective for the prevention of SSIs [10].

According to the latest evidence-based research, evidence of very low quality shows that it is uncertain whether antibiotics reduce the risk of postoperative wound infections after suture-based hernia repair, and evidence of moderate quality shows that antibiotics probably make little or no difference in preventing superficial or deep wound infections after mesh-type hernia repair in a low infection risk environment. Evidence of (very) low quality shows that antibiotics may reduce the risk of superficial wound infections but not deep wound infections after mesh-type hernia repair in a high infection risk environment [11]. In our case, an elderly patient with underlying diseases underwent bilateral inguinal hernia surgery, and the operation time was long. Therefore, multiple risk factors for wound infection coexisted.

The microbiology studies on SSIs have shown that most are caused by skin-derived bacteria such as *Staphylococcus aureus* and coagulase-negative staphylococci [12, 13]. These bacteria, particularly methicillin-resistant Staph aureus (MRSA) and MRCNS, are known for their ability to develop resistance to multiple antibiotics [14, 15]. To date, *Staphylococcus epidermidis* causes severe wound infection and is rarely reported clinically. The patient also tested positive for MRCNS, which belonged to multiple drug-resistant bacteria, and there were some difficulties in the selection of antibiotics.

Combined with the patient’s history of inguinal hernia without tension patch repair, wound redness and swelling, elevated inflammatory markers, and positive blood culture, wound infection after inguinal hernia repair can be clearly diagnosed. There are some controversies about which pathogen causes the wound infection. No positive bacteria were found in the secretion of the inguinal wound, while MRCNS was detected in blood culture, and *Acinetobacter lwoffi* was cultured from the pus in the left wrist. MRCNS is one of the main pathogenic bacteria of nosocomial infection, while *Acinetobacter lwoffi* is an opportunistic pathogen and is found widely in nature. The patient performed manual labor after surgery and had a history of contact with wood or unclean water at home, which infected the local skin of the left wrist, which was most likely caused by *Acinetobacter lwoffi*. Therefore, the pathogen causing inguinal wound infection was first considered MRCNS. In the selection of antibacterial drugs, we changed antibiotics many times according to clinical experience, while infection control was not ideal. According to the bacteriological drug sensitivity results, we selected antibacterial drugs sensitive to MRCNS and considered *Acinetobacter lwoffi*. Finally, the infection was effectively controlled. The patient’s initial selection of antibiotics was not sensitive to MRCNS, and the patient had a poor basic condition, poor nutritional status, hypoproteinemia, electrolyte disorder, and mild anaemia. This might be an important reason for the rapid progress of wound infection and aggravation of local and systemic infection symptoms.

The skin lesion of the infected wound in this patient was superficial, as described in the case description section. It needed to be distinguished from the following diseases: pyoderma gangraenosum, necrotizing fasciitis, and Meleney’s gangrene. (1) Painful erythema and nodular pustules appear in the early stage of pyoderma gangraenosum, which progress to multiple necrotic ulcers in a short period of time. The patches of purplish red along the edges were still growing when the scar healed at the centre of the ulcer. The disease might develop to some extent with bacterial infection, and the anti-infection treatment was not effective. Treatment with immunosuppressive drugs is required due to ulcers that were difficult to control [16]. Therefore, this diagnosis was not considered. (2) Necrotizing fasciitis can be divided into two types. Type I is a mixture of bacterial infections, including aerobic bacteria and anaerobic bacteria. Type II is mostly infected by a single Gram-positive bacterium, such as haemolytic Streptococcus and MRSA. The disease is more common in the limbs; the skin appears flaky red and swollen, with bloody blisters and severe pain in its early stages and subcutaneous superficial fascia extensive necrosis with tunnel infiltration in the later stages. As the disease progresses, the infection involves subcutaneous adipose tissue and deep muscles, symptoms of systemic poisoning could occur when local infection is mild, and sensory numbness occurs after local nerve involvement and destruction. As the disease develops rapidly, anti-infective therapy requires repeated surgical debridement [17]. In this case, we diagnosed it as inguinal wound infection. The infection involvement was superficial accompanied by local hyperalgesia, and it did not reach deep muscle tissue. Blood culture suggested *Staphylococcus epidermidis*, so we did not consider the disease. (3) Meleney’s gangrene occurs most often after trauma or surgery, mainly invading skin and subcutaneous tissue, and the clinical manifestations are superficial tissue necrosis, ulceration, and ulceration microvascular thrombosis. There is evidence of microaerobic bacteria, nonhaemolytic Streptococcus, and haemolytic *Staphylococcus*. Meleney believed that the synergistic effect of the two pathogenic bacteria in an anoxic environment was the main cause of ulcer progression and margin enlargement [18]. The wound infection in this patient was very similar to Meleney’s gangrene, and it also occurred after inguinal herniorrhaphy, while there was no evidence of multiple bacterial infections in the inguinal wound. Even so, the disease had not been ruled out. Simultaneous open repair of bilateral inguinal hernias is not encouraged as any complication on one side might occur on the other side as well, like in this case. This happens because the prevailing circumstances at the time of surgery are similar for both sides being operated. Therefore, laparoscopic inguinal hernia repair might be a better choice in bilateral inguinal hernia surgery based on the patient’s condition.

In conclusion, clinicians need to improve their awareness of wound infection after inguinal hernia surgery due to MRCNS. The patient’s condition should be comprehensively analysed before surgery for elderly patients with inguinal hernia, and individualized treatment is selected according to the patient’s situation to avoid or reduce the occurrence of SSIs. In our case, wound infection occurred after bilateral inguinal herniorrhaphy and developed rapidly, and the course of disease was long. Strengthening doctor-patient communication and establishing a good trusting relationship are the premises of successful treatment. Adequate drainage, and dressing changes of local wounds represent the basis of surgical treatment, and selecting the appropriate antibacterial drugs is the key to curing the disease according to the results of drug sensitivity analyses.

## Data Availability

All data and materials are presented in the manuscript.

## Abbreviations

MRCNS: Methicillin Resistant Coagulase Negative *Staphylococcus*
CT: Computed tomography
VAC: Vacuum assisted closure
SSIs: Surgical site infections
MRSA: Methicillin resistant *Staphylococcus aureus*
